# COVA1-18 neutralizing antibody protects against SARS-CoV-2 in three preclinical models

**DOI:** 10.21203/rs.3.rs-235272/v1

**Published:** 2021-02-15

**Authors:** P Maisonnasse, Y Aldon, A Marc, R Marlin, N Dereuddre-Bosquet, NA Kuzmina, AW Freyn, JL Snitselaar, A Gonçalves, TG Caniels, JA Burger, M Poniman, V Chesnais, S Diry, A Iershov, AJ Ronk, S Jangra, R Rathnasinghe, PJM Brouwer, TPL Bijl, J van Schooten, M Brinkkemper, H Liu, M Yuan, CE Mire, MJ van Breemen, V Contreras, T Naninck, J Lemaître, N Kahlaoui, F Relouzat, C Chapon, R Ho Tsong Fang, C McDanal, M Osei-Twum, N St-Amant, L Gagnon, DC Montefiori, IA Wilson, E Ginoux, GJ de Bree, A García-Sastre, M Schotsaert, L Coughlan, A Bukreyev, S van der Werf, J Guedj, RW Sanders, MJ van Gils, R Le Grand

**Affiliations:** 1 Université Paris-Saclay, Inserm, CEA, Center for Immunology of Viral, Auto-immune, Hematological and Bacterial diseases (IMVA-HB/IDMIT), Fontenay-aux-Roses & Le Kremlin-Bicêtre, Paris, France.; 2 Departments of Medical Microbiology of the Amsterdam UMC, University of Amsterdam, Amsterdam Institute for Infection and Immunity, 1105 AZ, Amsterdam, The Netherlands.; 3 Université de Paris, INSERM, IAME, F-75018 Paris, France.; 4 Department of Pathology, University of Texas Medical Branch at Galveston, Texas, USA; 5 Galveston National Laboratory, Texas, USA.; 6 Department of Microbiology, Icahn School of Medicine at Mount Sinai, New York (NY), USA.; 7 Life and Soft, 92350 Le Plessis-Robinson, France.; 8 Graduate School of Biomedical Sciences, Icahn School of Medicine at Mount Sinai, New York (NY), USA.; 9 Department of Integrative Structural and Computational Biology, The Scripps Research Institute, La Jolla, CA 92037, USA.; 10 Department of Microbiology, University of Texas Medical Branch at Galveston, Texas, USA.; 11 Duke Human Vaccine Institute & Department of Surgery, Durham, NC 27710, USA.; 12 Nexelis, Laval, Québec, Canada.; 13 Internal Medicine of the Amsterdam UMC, University of Amsterdam, Amsterdam Institute for Infection and Immunity, 1105 AZ, Amsterdam, The Netherlands.; 14 Department of Medicine, Division of Infectious Diseases, Icahn School of Medicine at Mount Sinai, New York (NY), USA.; 15 The Tisch Cancer Institute, Icahn School of Medicine at Mount Sinai, New York (NY), USA.; 16 Global Health and Emerging Pathogens Institute, Icahn School of Medicine at Mount Sinai, New York (NY), USA.; 17 University of Maryland School of Medicine, Department of Microbiology and Immunology and Center for Vaccine Development and Global Health (CVD), 685 W. Baltimore Street, HSF1, Office #380E, Baltimore, MD 21201.; 18 Molecular Genetics of RNA Viruses, Department of Virology, Institut Pasteur, CNRS UMR 3569, Université de Paris, Paris, France.; 19 National Reference Center for Respiratory Viruses, Institut Pasteur, Paris, France.; 20 Department of Microbiology and Immunology, Weill Medical College of Cornell University, New York, NY 10021, USA.

## Abstract

One year into the Coronavirus Disease 2019 (COVID-19) pandemic caused by Severe Acute Respiratory Syndrome coronavirus 2 (SARS-CoV-2), effective treatments are still needed^1-3^. Monoclonal antibodies, given alone or as part of a therapeutic cocktail, have shown promising results in patients, raising the hope that they could play an important role in preventing clinical deterioration in severely ill or in exposed, high risk individuals^4-6^. Here, we evaluated the prophylactic and therapeutic effect of COVA1-18 *in vivo*, a neutralizing antibody isolated from a convalescent patient^7^ and highly potent against the B.1.1.7. isolate^8,9^. In both prophylactic and therapeutic settings, SARS-CoV-2 remained undetectable in the lungs of COVA1-18 treated hACE2 mice. Therapeutic treatment also caused a dramatic reduction in viral loads in the lungs of Syrian hamsters. When administered at 10 mg kg^−1^ one day prior to a high dose SARS-CoV-2 challenge in cynomolgus macaques, COVA1-18 had a very strong antiviral activity in the upper respiratory compartments with an estimated reduction in viral infectivity of more than 95%, and prevented lymphopenia and extensive lung lesions. Modelling and experimental findings demonstrate that COVA1-18 has a strong antiviral activity in three different preclinical models and could be a valuable candidate for further clinical evaluation.

Across the world, the Coronavirus Disease 19 (COVID-19) pandemic caused by severe acute respiratory syndrome coronavirus 2 (SARS-CoV-2) continues to escalate^10^. Despite the progressive rollout of vaccines, there remains an urgent need for both curative and preventive measures, especially in individuals with high risk. Monoclonal neutralizing antibodies (NAbs), isolated from convalescent COVID-19 patients, are one of the most promising approaches and two NAb-based products have already received an emergency use authorization by the FDA. Although their clinical efficacy remains to be fully assessed^4-6^, their capability to reduce viral loads^4,5^ shows sufficient promise that such an approach could be effective if the treatment is administered early enough.

We and others have previously isolated and characterized several highly potent monoclonal NAbs with half-maximum inhibitory concentration (IC_50_) values in the picomolar range^7,11-14^, with the majority of these targeting the receptor binding domain (RBD) on the S1 subunit of the S protein. We previously identified COVA1-18, an RBD-specific monoclonal Ab, as one of the most potent NAb *in vivo*^7^. Using three different experimental models as well as mathematical modeling, we demonstrate that its rapid and extensive biodistribution is associated with a very potent antiviral effect, and make it a promising candidate for clinical evaluation, both as a prophylactic or therapeutic treatment of COVID-19.

## COVA1-18 *in vitro* potency is dependent on avidity

**Extended Data Figure 1.**
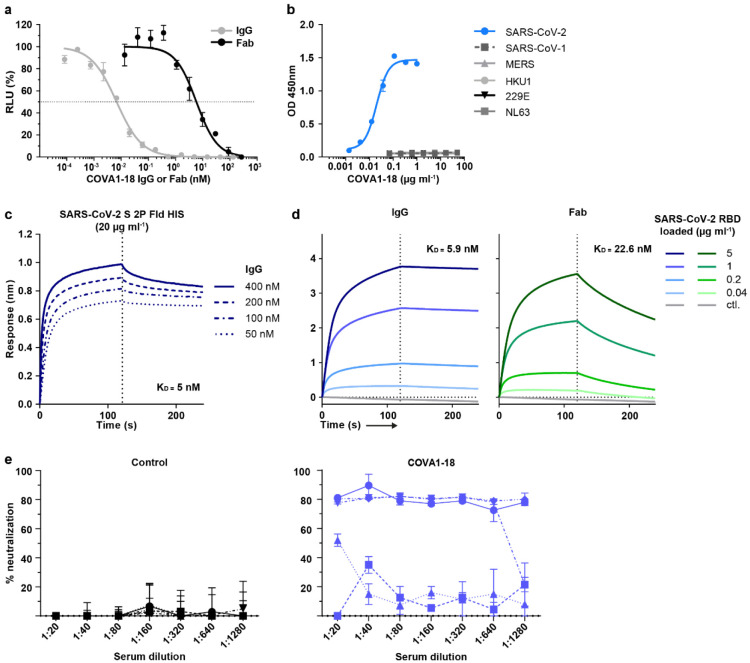
COVA1-18 IgG and Fab neutralization, cross-reactivity, binding kinetic and Syrian hamster serum neutralization. (**a**) IgG (grey) and Fab (black) pseudotype particle neutralization curves for COVA1-18. Representative of n ≥ 4 independent experiments. (**b**) Antigen specificity of COVA1-18 was assessed by ELISA against the soluble S protein derived from different human coronaviruses. (**c**) BLI sensorgrams of COVA1-18 binding to immobilized soluble SARS-CoV-2 S protein. Representative of n ≥ 2 independent experiments. (**d**) BLI sensorgrams of COVA1-18 binding to SARS-CoV-2 RBD loaded onto the sensor chip at various concentrations (n = 1). (**e**) Serum neutralization potency at 3 d.p.i. in Syrian hamsters for the control group (left) and COVA1-18 treated group (n = 5 animals per group).

To advance our earlier *in vitro* results^7^ on COVA1-18 and allow for better comparability with other studies, we used two new pseudovirus assays, one using lentiviral pseudotypes with an ACE2-expressing 293T cell line^15^, and one using VSV-pseudotypes with Vero E6 cells^16^, to confirm the potency of COVA1-18. With these assays, we confirmed the remarkable potency of COVA1-18 IgG which inhibited lentiviral SARS-CoV-2 pseudovirus with an IC_50_ of 0.8 ng ml^−1^ (5.2 pM) and VSV-based pseudovirus with an IC_50_ of 9 ng ml^−1^ (60 pM) (Extended Data Fig. 1a, Extended Data Table 1). These results were corroborated in multiple independent labs and COVA1-18 was also equipotent against the D614G variant (Extended Data Table 1) that now dominates worldwide^17-21^ as well as the recently emerged B.1.1.7 variant that includes the N501Y mutation^8,9^.

**Figure 1.**
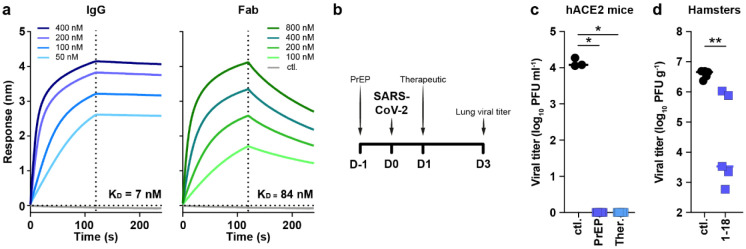
COVA1-18 avidity and SARS-CoV-2 protection in rodents. (**a**) Biolayer interferometry sensorgrams comparing COVA1-18 IgG and Fab binding to RBD. K_D_s are indicated. Representative of 3 independent experiments. (**b**) Study design with n = 5 per group, except mouse control group (n = 3). Hamsters were infected with 10^5^ PFU on day 0 and treated on day 1. Mice received COVA1-18 24 h prior to or after exposure to 10^4^ PFU. Lung viral titers at 3 d.p.i. are shown for mice (**c**) and hamsters (**d**). Bars indicate medians. Mann-Whitney unpaired t-test, *p* values: *<0.05, **<0.01. Ctl., control group; PrEP, pre-exposure prophylaxis; Ther., therapeutic.

COVA1-18 bound strongly to SARS-CoV-2 S protein and showed no cross-reactivity with S proteins of SARS-CoV, MERS-CoV and common cold coronaviruses HKU1-CoV, 229E-CoV and NL63-CoV (Extended Data Fig. 1b)^7^. Biolayer interferometry experiments showed that COVA1-18 IgG bound to soluble SARS-CoV-2 S protein with an apparent dissociation constant (K_D_) of 5 nM, and its affinity for RBD was similar at 7 nM (Fig. 1a, Extended Data Fig. 1c, d, Extended Data Table 1). Its Fab displayed a 12-fold weaker binding to RBD compared to IgG (84 nM), with the difference mainly caused by a faster Fab off-rate (Fig. 1a, Extended Data Table 1), also observed in a different assay setting (Extended Data Fig. 1d). With an IC_50_ of 199 ng ml^−1^, the COVA1-18 Fab was 237-fold less potent at neutralizing SARS-CoV-2 pseudovirus, showing that the IgG avidity effect is important for COVA1-18 neutralization potency (Extended Data Fig. 1a, Extended Data Table 1).

## COVA1-18 inhibits viral replication in rodents

We sought to evaluate whether COVA1-18 could control SARS-CoV-2 viral infection in a previously described Ad5-hACE2 mouse model^22,23^ using a 10 mg kg^−1^ dose. COVA1-18 administered intraperitoneally 24 h prior to, or after a SARS-CoV-2 challenge with 10^4^ plaque forming units (PFU) was fully protective with no detectable viral replication in the lungs (Fig. 1b, c). We then tested the efficacy of COVA1-18 in the golden Syrian hamster model (n = 5 per group), which is naturally susceptible to SARS-CoV-2 and develop severe pneumonia upon infection^24^. We evaluated the effect on lung viral loads of 10 mg kg^−1^ of COVA1-18 given 24 h after a 10^5^ PFU intranasal challenge (Fig. 1b, d). At 3 days post-infection (d.p.i.), 3/5 animals had high serum neutralization while 2/5 animals had low neutralization activity (Extended Data Fig. 1e). On day 3, the COVA1-18 treated group had significantly lower median lung viral titers compared to the control group (3.5 vs 6.7 log_10_ PFU g^−1^, respectively, *p*0.01) with lowest viral titers in the higher serum neutralizers (Fig. 1d).

## COVA1-18 PrEP prevents infection in NHP

**Extended Data Figure 2.**
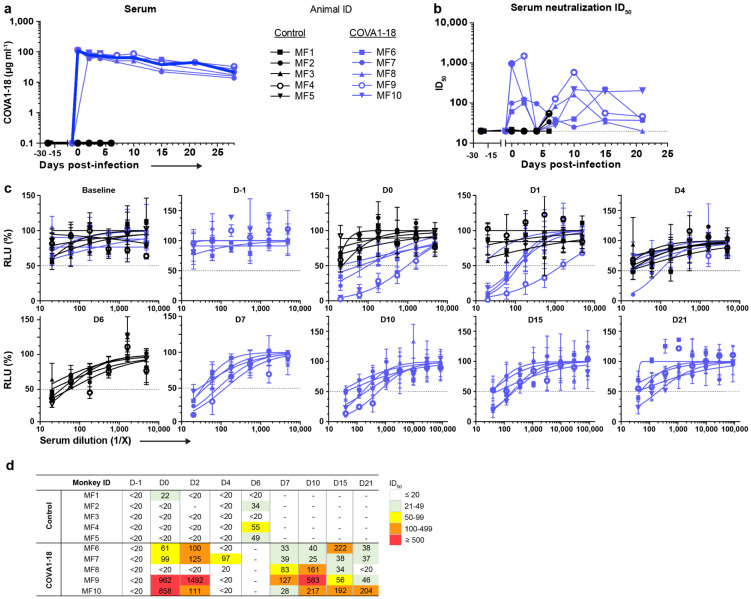
Serum and mucosal pharmacokinetics of COVA1-18 in treated macaques (1/2). (**a**) Serum COVA1-18 concentration for each animal. The mean COVA-18 concentration for each group is indicated by a thick blue line (treated animals) and a thick black line (control). (**b**) Individual serum neutralization ID_50_. (**c**) Serum neutralization curve for each animal at the indicated day post-treatment. (**d**) Individual serum neutralization ID_50_ with titer range indicated as ID_50_ 21-49 in green, 50-99 in yellow, 100-499 in orange, >500 red.

**Extended Data Figure 3.**
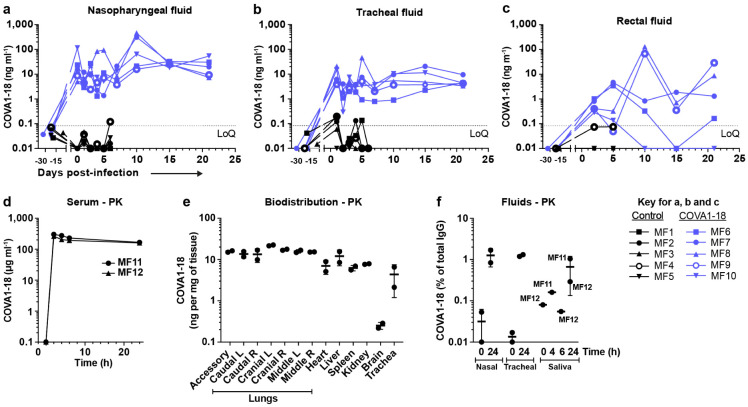
Serum and mucosal pharmacokinetics of COVA1-18 in treated macaques (2/2). The COVA1-18 concentrations measured in nasopharyngeal (**a**), tracheal (**b**) and rectal (**c**) fluids by ELISA are reported for each animal in both groups. (**d**) Serum COVA1-18 concentration from two animals injected with 10 mg kg^−1^ of COVA1-18 and sampled at 0, 2, 4, 6 and 24 h for a pharmacokinetic (PK) study. (**e**) The two macaques were euthanized at 24 h post-treatment and organs analyzed to assess the biodistribution of COVA1-18. The concentration of COVA1-18 was normalized to the weight of each sample for every organ. (**f**) COVA1-18 was measured in fluid samples of the PK study animals and normalized to the total cynomolgus IgG content for each sample. LoQ, limit of quantification.

**Figure 2.**
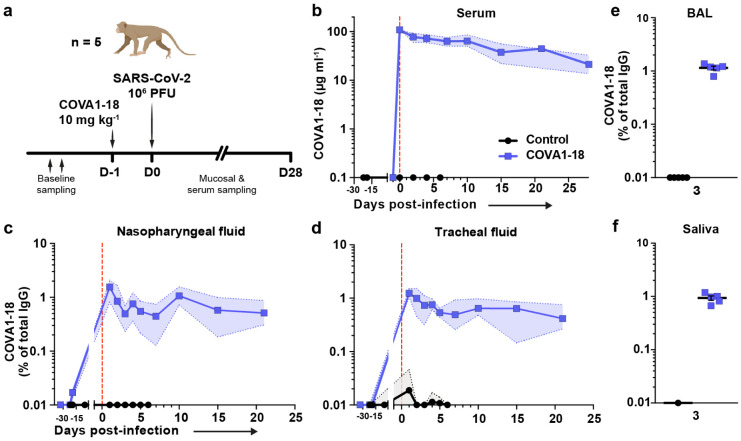
COVA1-18 serum and mucosal pharmacokinetic in infected cynomolgus macaques. (**a**) Study design. Two groups of n = 5 were exposed to 10^6^ PFU of SARS-CoV-2 (intranasal and intratracheal routes). Treated animals received 10 mg kg^−1^ of COVA1-18 1 day before challenge. (**b**) COVA1-18 serum concentration (mean with range). COVA1-18 concentration reported as percent of total cynomolgus IgG in heat-inactivated (**c**) nasopharyngeal fluid, (**d**) tracheal fluid (means with range), (**e**) bronchoalveolar lavage (BAL) and (**f**) saliva (means ± SEMs). The red dashed line indicates challenge day.

We evaluated the potential of COVA1-18 to prevent SARS-CoV-2 infection in cynomolgus macaques in a pre-exposure prophylaxis (PrEP) study. The animals were treated intravenously 24 h prior to viral challenge with a dose of 10 mg kg^−1^ of COVA1-18 (Fig. 2a). Treated and control animals (n = 5 per group) were challenged on day 0 with 10^6^ PFU of SARS-CoV-2 via combined intranasal and intratracheal routes using an experimental protocol developed previously^25,26^. On the day of challenge, the mean COVA1-18 serum concentration was 109 ± 2.7 μg ml^−1^ (Fig. 2b, Extended Data Fig. 2a), and 4/5 animals had serum neutralization activity while no neutralization activity was observed in the control group (Extended Data Fig. 2b-d). COVA-18 was also detected in all respiratory tract samples and rectal samples (Fig. 2c-e, Extended Data Fig. 3a-c), and represented on average 1.5% and 1.2% of the total IgG in heat-inactivated content in the nasopharyngeal and tracheal mucosae, respectively. These levels remained constant throughout the study period and similar levels were detected at 3 d.p.i. in bronchoalveolar lavages (BAL) and saliva (Fig. 2e-f). As SARS-CoV-2 can cause damage to non-respiratory organs, we performed a pharmacokinetic study on two additional macaques to characterize the COVA1-18 distribution within the first 24 h (Extended Data Fig. 3d-f). COVA1-18 was found in all organs studied, including the lungs, at concentrations of 4 to 22 ng mg^−1^ of tissue, except for the brain where concentrations where substantially lower (250 pg mg^−1^ of tissue). Altogether, these data showed that COVA1-18 administered intravenously was rapidly and efficiently distributed to the natural sites of infection as well as to organs affected by COVID-19 pathology.

**Extended Data Figure 4.**
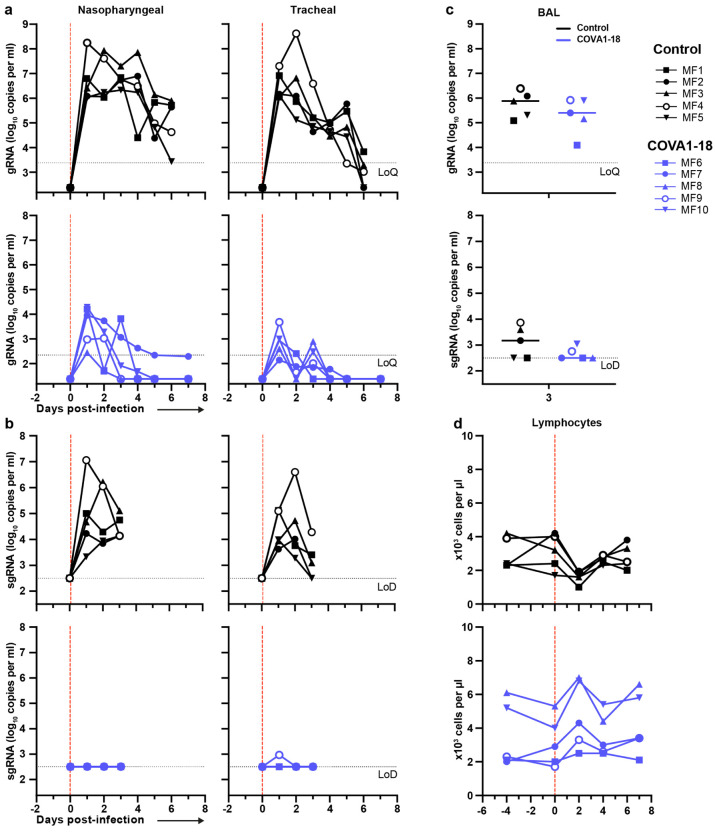
COVA1-18 pre-exposure prophylaxis protects cynomolgus monkeys against SARS-CoV-2 challenge and clinical symptoms. (**a**) Genomic (g)RNA and (**b**) subgenomic (sg)RNA loads determined by PCR in nasopharyngeal fluids (left) and tracheal fluids (right) of control (top) and treated (bottom) animals. (**c**) gRNA (top) and sgRNA (bottom) in the bronchoalveolar lavages (BAL) at day 3 post-infection. (**d**) Absolute lymphocyte count in the blood of control (top) and treated (bottom) animals. LoD, limit of detection; LoQ, limit of quantification.

**Figure 3.**
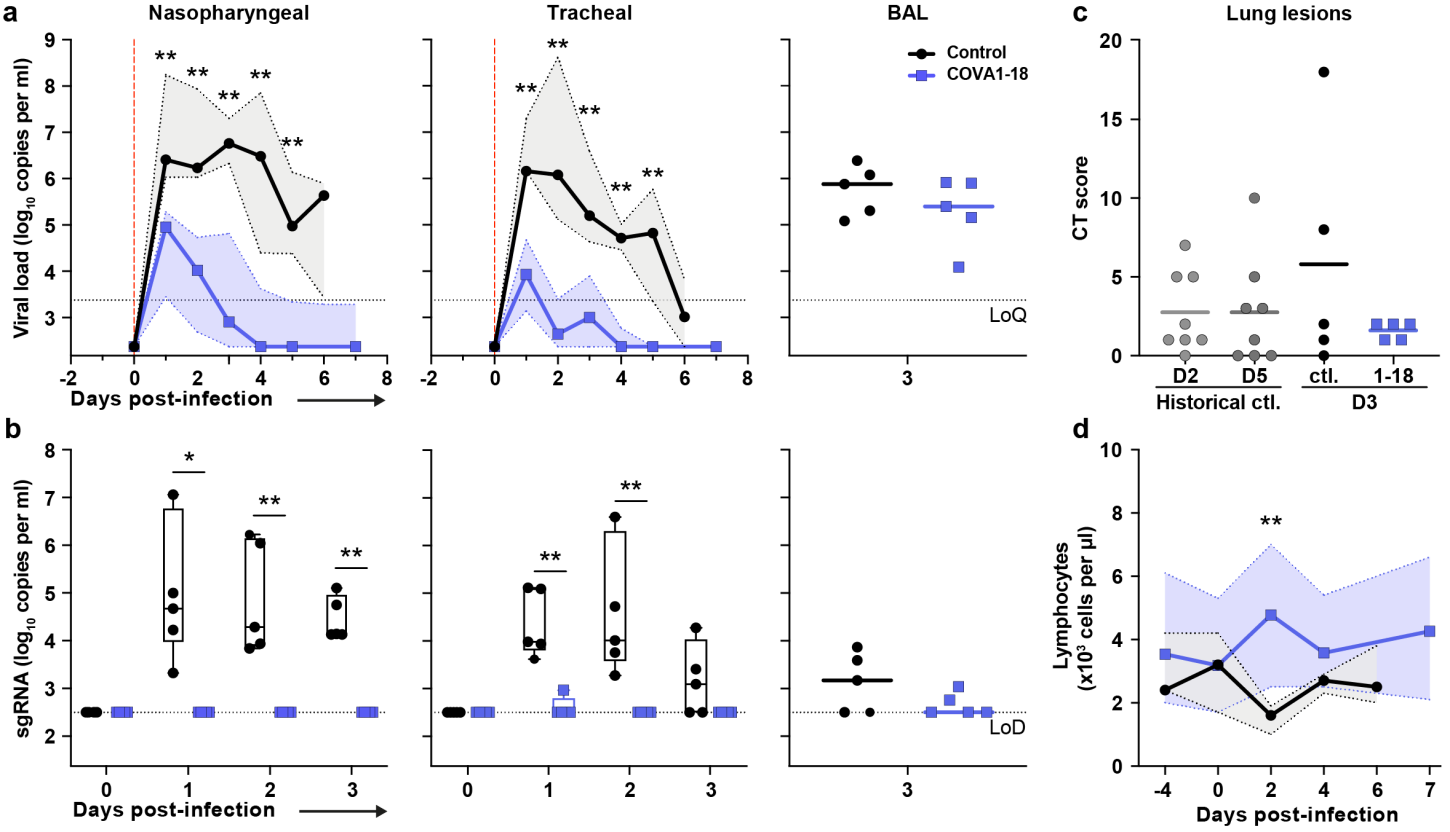
COVA1-18 pre-exposure prophylaxis protects cynomolgus monkeys against SARS-CoV-2 challenge and clinical symptoms. (**a**) Genomic (g)RNA and (**b**) subgenomic (sg)RNA loads determined by PCR in nasopharyngeal fluids (left), tracheal fluids (middle) and bronchoalveolar lavages (BAL) (right). Medians with range are indicated for fluids and bars represent medians for BAL. (**c**) Chest CT scores were determined at 3 d.p.i. and at 2 or 5 d.p.i for historical controls. (**d**) Absolute lymphocyte count in the blood (mean with range). Mann-Whitney unpaired t-test, *p* values: * < 0.05, ** < 0.01. Ctl., control group; LoD, limit of detection; LoQ, limit of quantification.

Following viral challenge, control animals showed similar genomic (g)RNA and subgenomic (sg)RNA levels and kinetics as previously described^25,26^ with median peak viral loads (VL) of 6.4 and 6.2 log_10_ copies per ml at 1-2 d.p.i. in the nasopharyngeal and tracheal swabs, respectively (Fig. 3a and Extended Fig. 4a). Active viral replication, as assessed by sgRNA levels, peaked at 1-2 d.p.i. in nasopharyngeal and tracheal swabs with median values of 4.6 and 4.0 log_10_ copies per ml, respectively (Fig. 3b and Extended Fig. 4b). At 3 d.p.i., viral loads were detected in the BAL with a median value of 4.9 log_10_ copies per ml of gRNA and 3.2 log_10_ copies per ml of sgRNA, including 3 animals with no detectable sgRNA.

In comparison, treated animals had a reduction of 2.2 and 3.4 log_10_ median gRNA VL in tracheal swabs on days 1 and 2 (both *p*<0.01 to controls), and had undetectable VL after day 4 (Fig. 3a and Extended Fig. 4a). The difference was also evident in nasopharyngeal swabs, with treated animals having a reduction of 1.5 and 2.2 log_10_ gRNA VL on days 1 and 2 (both *p*<0.01 to controls). By day 4, 4/5 treated animals had undetectable gRNA in the nasopharyngeal swabs while one animal (MF7) remained positive with a low residual gRNA signal up to 7 d.p.i.. COVA1-18 treatment dramatically hindered viral replication in the upper respiratory tract as evidenced by the absence of detectable sgRNA in the nasopharyngeal and tracheal swabs for all treated animals with the exception of animal (MF9) that showed a low signal at 1 d.p.i. only in the tracheal swabs (Fig. 3b and Extended Fig. 4b). Therefore, in the treated group, most upper respiratory tract gRNA VL likely represents the progressive elimination of the challenge inoculum, and does not result from active replication. The gRNA and sgRNA loads in BAL were also lower in COVA1-18 recipients compared to controls but the difference did not reach statistical significance (Fig. 3a, b, Extended Fig. 4c). Overall, these results demonstrate that a 10 mg kg^−1^ dose of COVA1-18 PrEP dramatically reduced the acquisition and/or early spread of SARS-CoV-2 in the different respiratory compartments.

Analysis of lung lesions by chest computed tomography (CT) showed that all treated animals had few and small lung lesions as recorded by low CT scores at 3 d.p.i. while 2/5 controls showed mild pulmonary lesions characterized by non-extended ground-glass opacities (GGOs) with scores superior to 5, consistent with what was observed in historic controls (Fig. 3c)^25^. In addition, we observed that all control animals were lymphopenic at 2 d.p.i., consistent with previous studies^25,26^, while all treated animals had normal lymphocyte counts throughout the study (*p*<0.01 for the comparison) (Fig. 3d, Extended Fig. 4d).

**Extended Data Figure 5.**
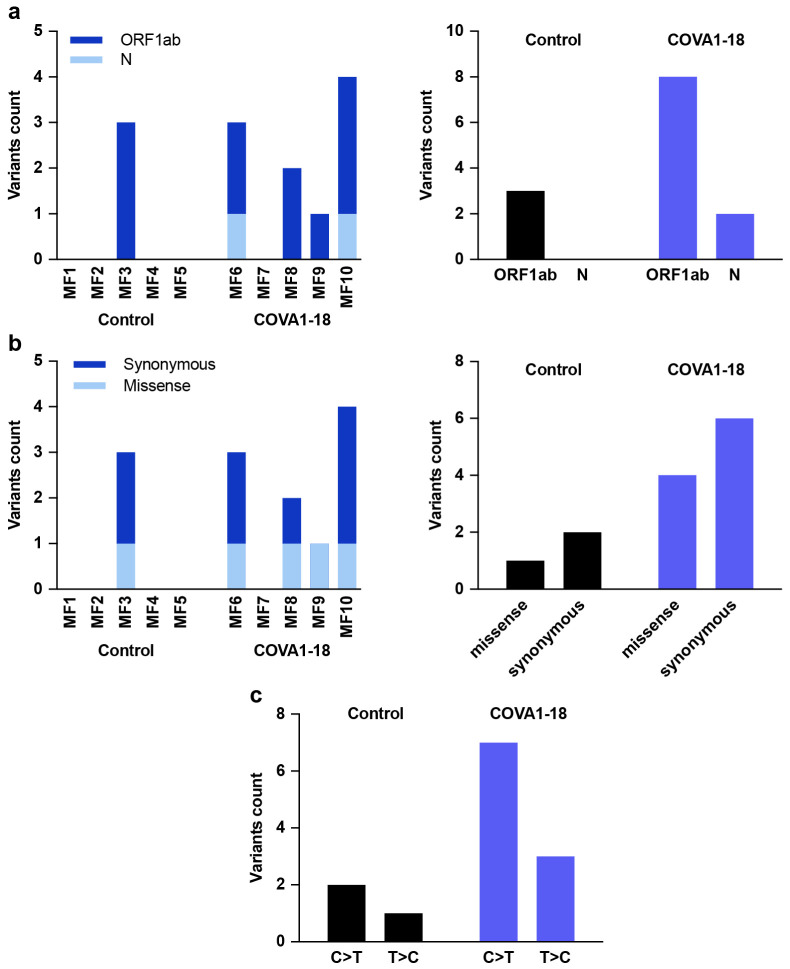
Sequences in treated and exposed NHP. Viral population sequences in the nasopharyngeal swabs at day 3 were analyzed by Next Generation Sequencing. (**a**) Variants count detected in the N and ORF1ab genes for each individual (left) and cumulative variants count for each gene in the control and COVA1-18 treated groups (right). (**b**) Individual (left) and cumulative (right) synonymous and missense variants count for the control and treated groups. (**c**) Nucleotide substitution observed by type for both groups.

One concern about SARS-CoV-2 vaccines and NAb treatments is the possible generation of suboptimal concentrations of NAb in individuals, which could foster viral escape^27^. The COVA1-18 treatment resulted in enrichment of subclonal variations in N and ORFlab, but no treatment-induced escape mutations were detected in the *S* gene when applying standard quality filters (Extended Data Fig. 5).

## Prediction models refine COVA1-18 dosage

**Extended Data Figure 6.**
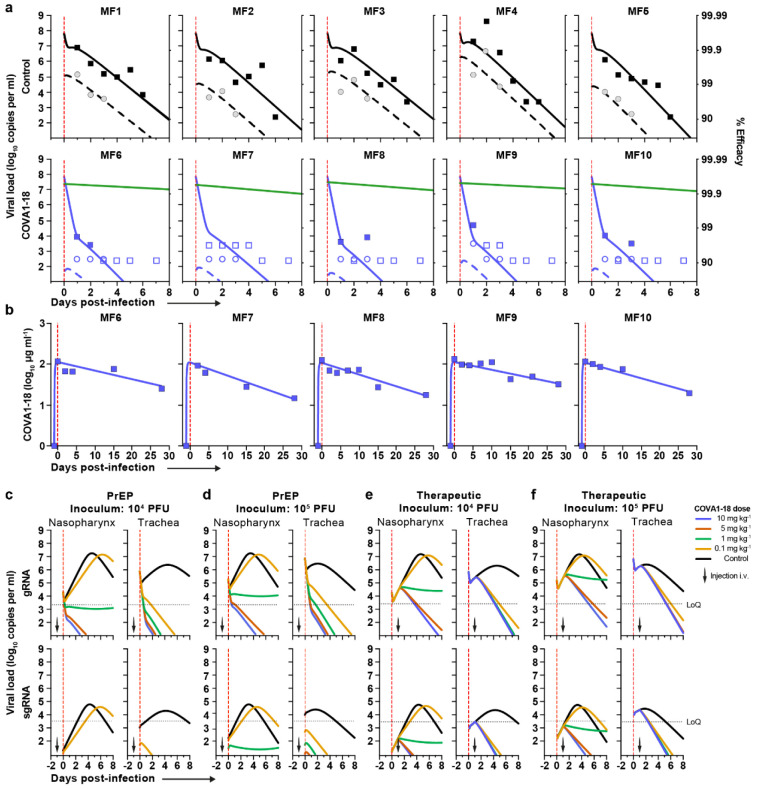
Modeling of viral dynamics and treatment efficacy (1/2). (**a**) Individual prediction of the tracheal gRNA and sgRNA in control (top) and treated animals (bottom) with individual efficacy prediction indicated (green line). The dashed red line indicates the time of viral infection. gRNA (squares) and sgRNA (circles) data are indicated as plain (above LoQ) or open (below LoQ). (**b**) Individual prediction of the COVA1-18 plasma concentration. (**c-d**) Simulation of the predicted gRNA (top) and sgRNA (bottom) viral loads in the nasopharynx and trachea for a 10^4^ and 10^5^ PFU challenge dose according to the dose of COVA1-18 given 24 h prior challenge (arrow). (**e-f**) Simulation as in (**c**) with COVA1-18 given 24 h post-infection. Black dotted lines indicate the limit of quantification (LoQ). i.v., intravenous; PFU, plaque forming units. PrEP, Pre-Exposure Prophylaxis.

**Figure 4.**
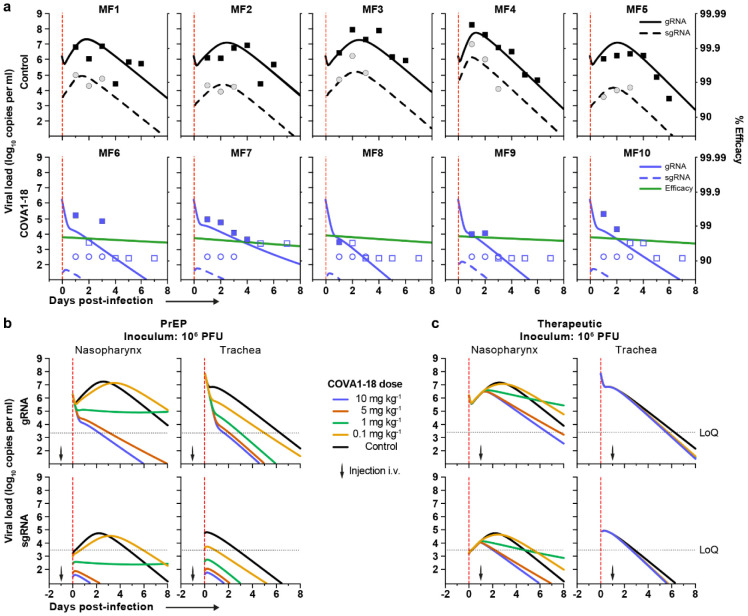
Modeling of viral dynamics and treatment efficacy. (**a**) Individual prediction of the nasopharyngeal gRNA and sgRNA in control (top) and treated animals (bottom) with individual efficacy prediction indicated (green line). The dashed red line indicates the time of infection. gRNA (squares) and sgRNA (circles) data are indicated as plain (above LoQ) or open (below LoQ). (**b**) Model predictions of gRNA and sgRNA dynamics with 4 doses of COVA1-18 given 24 h prior to challenge (arrow). (c) Simulation as in (b) with COVA1-18 given 24 h post-infection. Black dotted lines indicate LoQ (limit of quantification); i.v., intravenous; PFU, plaque forming units.

Next, we used a viral dynamic model previously developed in the same SARS-CoV-2 NHP experimental model^28^ to evaluate the level of protection conferred by COVA1-18. The model considers a target cell limited infection in both nasopharyngeal and tracheal compartments. In addition to the previously developed model, we assumed that sgRNA was a proxy for the total number of non-productively and productively infected cells (see supplementary methods) and we further assumed that COVA1-18 plasma drug concentrations over time, noted C(t), was the driver of drug efficacy. We modeled the changes in C(t) using a standard first order absorption and elimination model, which led estimated half-life of COVA1-18 in plasma of 12.6 days (Extended Data Fig. 6b). We assumed that COVA1-18 reduces infectivity rate in both tracheal and nasopharyngeal compartments with an efficacy, noted *η*(*t*), determined by the following model $\eta (t)=\frac{C(t)}{C(t)+EC_{50}}$, where EC_50_ is the plasma COVA1-18 concentrations corresponding to a 50% reduction of viral infectivity. The model fitted the viral kinetics well in all animals (Fig. 4a, Extended Data Fig. 6a, Extended Data Table 2). In treated animals, *EC*_50_ was estimated to 2.2 and 0.053 μg ml^−1^ in the nasopharynx and trachea, respectively, which is roughly 50 and 2000 times lower than the plasma drug concentrations of 109.7 μg ml^−1^ observed on the day of infection (see above). Thus these results confirm that the efficacy of COVA1-18 was very high, with efficacies above 95% and 99.9% in nasopharyngeal and tracheal compartments on the day of infection, respectively (Fig. 4a, Extended Data Fig. 6a) Given the long half-life of the drug, this efficacy could be maintained over time, and we estimated that the mean individual efficacy of the COVA1-18 in the first 10 days following infection ranged between 96.67% and 97.50% in the nasopharynx and between 99.91% and 99.94% in the trachea (Extended Data Table 3).

**Extended Data Figure 7.**
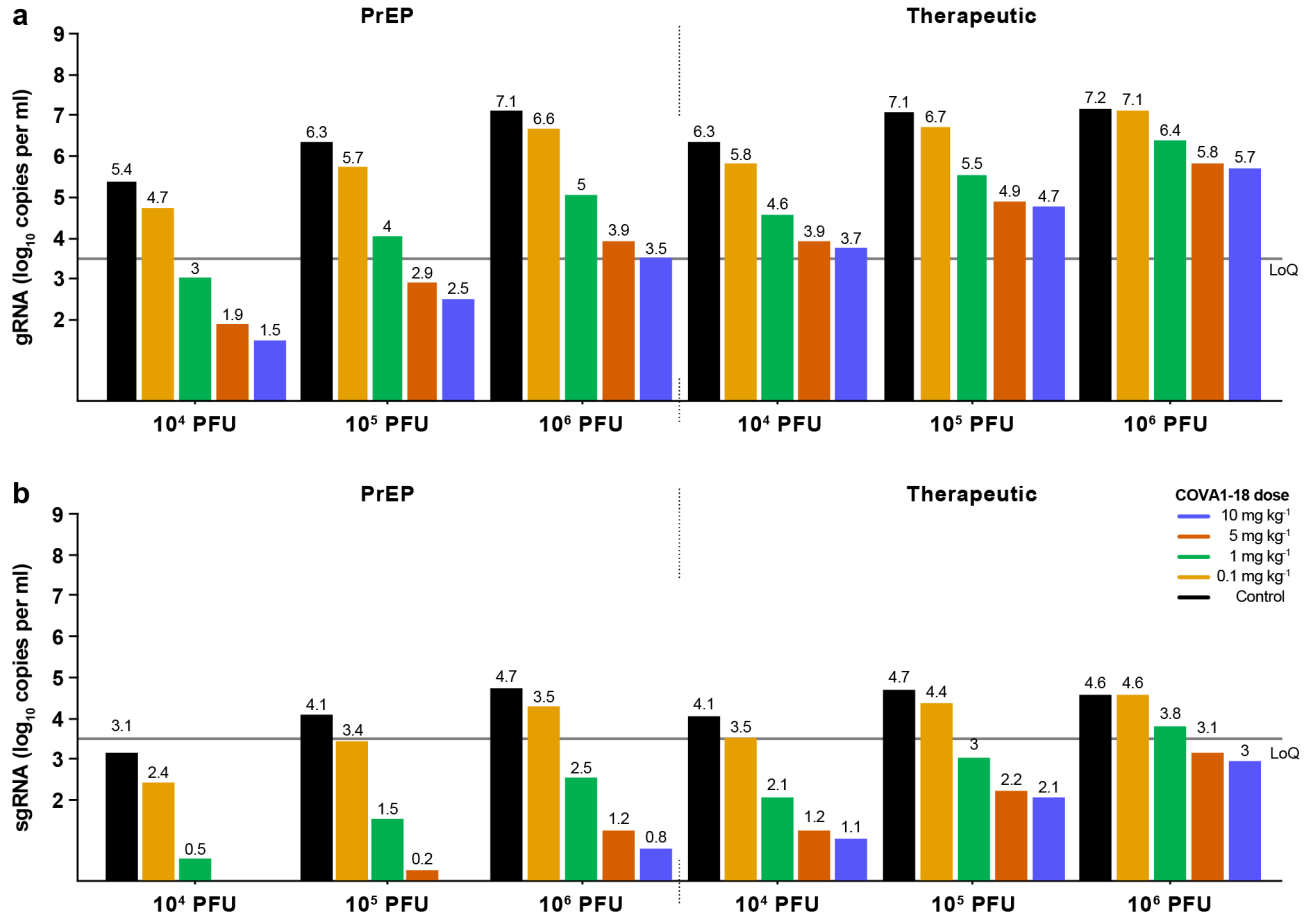
Modeling of viral dynamics and treatment efficacy (2/2). Simulation of the predicted gRNA (top) and sgRNA (bottom) viral loads in the nasopharynx, according to the dose of COVA1-18 received and the dose of virus received. Left: Pre-Exposure Prophylaxis (PrEP) treatment at −1 d.p.i., viral load measured at 2 d.p.i.; Right: Therapeutic treatment at 1 d.p.i., viral load measured at 3 d.p.i. Black: control; yellow: 0.1 mg kg^−1^; green: 1 mg kg^−1^: orange: 5 mg kg^−1^; blue: 10 mg kg^−1^. LoQ, limit of quantification.

Next, we used our model to investigate changes in experimental conditions, such as COVA1-18 dose being administered at a lower dose and/or after the viral challenge (see methods). In all scenarios considered, a dose of 5 mg kg^−1^ was determined to provide nearly similar results than 10 mg kg^−1^ (Fig. 4b, c, Extended Data Fig. 7). A dose of 1 mg kg^−1^ could be sufficient to prevent active viral replication as long as treatment is given prior to infection, but might be insufficient in a therapeutic setting. However, this dose could be relevant if lower doses of virus were used for infection, such as 10^4^ or 10^5^ PFU (Extended Data Fig 6c-f).

## Discussion

Despite the recent approval of several SARS-CoV-2 vaccines by health authorities, the slow roll-out of vaccination campaigns will not result in resolution of the pandemic in the immediate future. Furthermore, the emergence of viral escape mutants may lead to reduced vaccine efficacy, and some individuals, such as immunocompromised patients or the elderly, may not mount adequate protective immune responses to vaccination. Thus, there is an urgent need to develop effective therapeutics, in particular for individuals with high risk of severe disease. Pre-clinical and clinical studies to evaluate SARS-CoV-2 NAbs for prophylaxis and/or treatment and such studies have supported the implementation of several NAb candidates and NAb cocktails for emergency use^22,29-35^. However, the narrow efficacy range of FDA-approved NAbs ^4-6^, together with rapidly spreading new variants complicate treatment strategies^36-39^, highlights the need for additional treatment options, including potent NAbs such as COVA1-18.

In hACE2-expressing mice and golden Syrian hamsters, COVA1-18 showed remarkable control of SARS-CoV-2 infection. These promising results were confirmed in NHPs, with COVA1-18 given one day prior to infection achieving nearly complete protection in the upper respiratory tract in cynomolgus macaques. Using a viral dynamic model, we estimated that COVA1-18 reduced viral infectivity by >95% and 99.9% in nasopharyngeal and tracheal compartments, respectively. The robustness of these results are reinforced by the high challenge dose that we used, which was 10 to 100-fold higher than in other NHP studies evaluating NAbs for PrEP against SARS-CoV-2^29-34^. In fact, the model allowed us to predict, without using additional animals, that protection could be achieved with lower doses of 5 mg kg^−1^ and 1 mg kg^−1^ with an inoculum dose of 10^5^ or 10^4^ PFU, both in prophylactic and therapeutic settings (Extended Data Fig. 6, Extended Data Fig. 7).

How do these levels of efficacy greater than 95% translate into clinical efficacy? In previous work, we estimated that achieving 90% efficacy would be sufficient to confer a high level of protection against infection acquisition if treatment can be administered prophylactically or just after a high-risk contact^40^. In hospitalized patients, where viral load kinetics after admission is associated with the risk of death, we estimated that administration of treatment with an efficacy higher than 90% could reduce the time to viral clearance by more than 3 days in patients over 65 years of age, which could translate into significantly lower rates of mortality in this population^41^. Altogether, the results obtained here in a NHP model suggest that COVA1-18 could be a valuable candidate for clinical evaluation.

A relevant concern is that these results may be jeopardized by the increasing prevalence of mutant strains, which could reduce the sensitivity to NAbs. While escape mutations can arise following single NAb treatment as recently demonstrated^31,42^, COVA1-18 did not select for S protein escape mutants when evaluated as PrEP in NHP. Importantly, studies have determined that COVA1-18 retains high potency against the B.1.1.7 variant, which includes the N501Y mutation^8,9^. However, as it is derived from IGHV3-66, it will likely lose potency against variants harboring the E484K mutation (*i.e.* the B.1.351 and B.1.1.28 lineages), as recently shown for convalescent plasma and many NAbs^37,38^. This highlights the necessity of using NAbs cocktails targeting distinct epitopes. In addition, the half-life of COVA1-18 can be extended by incorporating the LS or YTE^43^ mutations which can further reduce the protective dose required and reduce the cost of treatment.

In conclusion, our COVA1-18 *in vitro* data translated into a powerful protective drug in three preclinical models to prevent SARS-CoV-2 replication. Together with our prediction model, these data showed that COVA1-18 could be used in patients at low doses either to prevent infection or to reduce viral loads in a therapeutic setting, with a potential greater impact in high-risk patients. The high *in vivo* efficacy of COVA1-18 and its demonstrated potency against the B.1.1.7. isolate also suggests it is a great candidate for a NAb cocktail.

## Methods

### IgG, Fab and soluble viral protein expression

COVA1-18 was isolated from a participant in the “COVID-19 Specific Antibodies” (COSCA) study as described^7^. The COSCA study was conducted at the Amsterdam University Medical Centre, location AMC, the Netherlands and approved by the local ethical committee of the AMC (NL 73281.018.20). COVA1-18 IgG was produced in HEK293F suspension cells as previously described^7^. COVA1-18 His-tagged Fab was produced in ExpiCHO cells as previously described^44^. Spike and RBD proteins were produced and purified as previously described^7^.

### Bio-layer interferometry

The affinity of COVA1-18 IgG and His-tagged Fab versions were determined using Ni-NTA biosensors (ForteBio) onto which 20 μg ml^−1^ of SARS-CoV-2 RBD in running buffer (PBS, 0.02% Tween-20, 0.1% BSA) was loaded for 300 s as previously described^44^. The association rate and dissociation step were assessed over a 120 s step each. Serially diluted IgG (50, 100, 200 and 400 nM) and Fab (100, 200, 400 and 800 nM) were tested and an anti-HIV-1 His-tagged Fab at 800 nM in running buffer was included as negative control. K_D_s were determined using ForteBio Octet CFR software using a 1:2 fitting model for IgGs and a 1:1 fitting model for Fabs. The apparent affinity of COVA1-18 IgG to the SARS-CoV-2 S trimer was determined as described above except that 20 μg ml^−1^ SARS-CoV-2 S 2P Fid His protein was loaded instead of RBD. The COVA1-18 IgG avidity effect was further evaluated by titrating the loaded SARS-CoV-2 RBD (5, 1, 0.2 and 0.04 μg ml^−1^). An additional loading step using His-tagged HIV-1 gp41 was performed to minimize background binding of His-tagged Fabs to the biosensor and both the COVA1-18 IgG and Fab concentrations were set at 250 nM. All other steps were performed as described above.

### Ni-NTA-capture ELISA

SARS-CoV-2, SARS-CoV, MERS, HKU1, 229E and NL63 S His-tagged proteins were loaded at 2 μg ml^−1^ in TBS/2% skimmed milk (100 μl/well) on 96-well Ni-NTA plates (Qiagen) for 2 h at room temperature (RT). Three-fold serially diluted COVA NAb were then added onto the plates for 2 h at RT followed by the addition goat anti-human IgG-HRP (Jackson Immunoresearch) secondary Ab (1:3000) for 1 h at RT. The plates were developed for 3 min using TMB solution then stopped, optical densities measured at 450 nm on a spectrophotometer and data graphed using GraphPad Prism software (v8.3.0).

### Detection of human IgG in NHP fluid

Detection of COVA1-18 in NHP samples determined by ELISA using a protocol adapted from others^30^. Briefly, half area high binding 96-well plates (Greiner Bio-One) were coated overnight with goat anti-Human IgG H+L (monkey pre-adsorbed) at 1 μg ml^−1^ in PBS. The plates were then blocked in casein buffer (Thermo Scientific) for 2 h at RT. Serum and mucosal samples were serially diluted and loaded onto the plates as well as serially diluted COVA1-18 as the standard. Following a 1 h RT incubation, goat anti-Human IgG (monkey adsorbed)-HRP secondary antibody (Southern Biotech) was added for serum samples (1:4000). For mucosal samples, goat anti-Human IgG (monkey adsorbed)-BIOT (Southern Biotech) was added at 1:10000 dilution. After 1 h RT incubation, serum sample plates were ready for development. For mucosal samples, an additional 1 h incubation with poly-HRP40 (Fitzgerald) (1:10000) was necessary. The plates were then developed for 5 min, the optical densities measured at 450 nm on a spectrophotometer and raw data exported and analyzed using Microsoft Excel and GraphPad Prism (v8.3.0) software. The COVA1-18 concentration in a specific sample was determined by interpolating OD values from dilutions that fell into the linear range of the standard curve of the matching ELISA plate.

### Cynomolgus monkey IgG ELISA

Half area high binding 96-well plates were coated overnight (4 °C) with goat anti-Human IgG λ and goat anti-Human IgG κ (Southern Biotech), 1:2000 (each) in PBS, 50 μl/well. The plates were washed (IX TBS–0,05% Tween20) and block for 2 h at RT with 50 μl/well casein buffer. Serially diluted mucosal and serum samples were loaded onto the plates. Serially diluted polyclonal cynomolgus IgG (Molecular Innovations) was used as standard. Following a 1 h incubation at RT, mouse anti-Monkey IgG Fc-BIOT (Southern Biotech) was loaded onto the plate (1:50000). After 1 h at RT, poly-HRP40 was added (1:10000) and the plates incubated for 1 h. Finally, the plates were washed 5 times, developed for 5 min, and analysed as described above.

### Pseudovirus neutralization assay

Neutralization assays were performed using SARS-CoV-2 S-pseudotyped HIV-1 virus and HEK293T hACE2 cells as described previously^15^. In brief, pseudotyped virus was produced by co-transfecting expression plasmids of SARS-CoV-2_Δ19_ S proteins (GenBank MT449663.1) with an HIV backbone expressing NanoLuc luciferase (pHIV-1_NL4-3_ ΔEnv-NanoLuc) in HEK293T cells (ATCC, CRL-11268). After 2 days, the cell culture supernatants containing SARS-CoV-2 S-pseudotyped HIV-1 viruses were harvested and stored at −80 °C. HEK293T hACE2 cells were seeded 20,000 cells/well in a flat-bottom 96-well plates one day prior to the start of the neutralization assay. COVA1-18 IgG and His6-tagged Fab as well as heat-inactivated serum samples were serially diluted in 3-fold steps using cell culture medium and then mixed with pseudotyped virus in a 1:1 ratio and incubated for 1 h at 37 °C. The mixtures were then added to the HEK293T hACE2 cells in a 1:1 medium to mixture ratio. The final starting concentration for IgGs was 20 μg ml^−1^ and 13.33 μg ml^−1^ for Fab. The cells were then incubated at 37 °C for 48 h followed by one PBS wash and lysis buffer addition. The luciferase activity in the cell lysates was measured using the Nano-Glo Luciferase Assay System (Promega) and GloMax Discover microplate reader. Relative luminescence units (RLU) were normalized to those from positive control wells where cells were infected with SARS-CoV-2 pseudovirus without IgG, Fab or serum. The inhibitory concentration (IC_50_) and neutralization titers (ID_50_) were determined as the IgG/Fab concentration or serum dilution at which infectivity was inhibited by 50%.

Pseudotyped Vesicular Stomatitis Virus (VSVΔG) particles displaying SARS-CoV-2_Δ19_ S and containing a luciferase reporter were used as previously described^16^. Two-fold dilution series of COVA1-18 were prepared in complete medium, pseudotyped virus added and the mixture incubated for 1 h at 37 °C. The virus-antibody mixtures were then loaded onto plates seeded with Vero E6 cells 24 h prior this step. Following a 20 h incubation 37 °C, the luciferase substrate was added to lysed cells and RLU determined and analyzed as described above.

### Ethics and biosafety statement

Female golden Syrian hamsters, aged 6-7 weeks, were housed in the ABSL-4 facility of the Galveston National Laboratory. The animal protocol # 2004049 was approved by the Institutional Animal Care and Use Committee (IACUC) of the University of Texas Medical Branch at Galveston (UTMB).

The mouse experimental study was approved by the Icahn School of Medicine at Mount Sinai Institutional Animal Care and Use Committee (IACUC-2017-0170 and IACUC-2017-0330). Male and female cynomolgus macaques (*Macaca fascicularis*), aged 3-6 years and originating from Mauritian AAALAC certified breeding centers were used in this study. All animals were housed in IDMIT infrastructure facilities (CEA, Fontenay-aux-roses), under BSL-2 and BSL-3 containment when necessary (Animal facility authorization #D92-032-02, Préfecture des Hauts de Seine, France) and in compliance with European Directive 2010/63/EU, the French regulations and the Standards for Human Care and Use of Laboratory Animals, of the Office for Laboratory Animal Welfare (OLAW, assurance number #A5826-01, US). The protocols were approved by the institutional ethical committee “Comité d’Ethique en Expérimentation Animale du Commissariat à l’Energie Atomique et aux Energies Alternatives” (CEtEA #44) under statement number A20-011. The study was authorized by the “Research, Innovation and Education Ministry” under registration number APAFIS#24434-2020030216532863.

### Ethics committee

All information on the ethics committee is available at https://cache.media.enseignementsup-recherche.gouv.fr/file/utilisation_des_animaux_fins_scientifiques/22/1/comiteethiqueea17_juin2013_257221.pdf

### Viruses and cells

For the macaques studies, SARS-CoV-2 virus (hCoV-19/France/ 1DF0372/2020 strain) was isolated by the National Reference Center for Respiratory Viruses (Institut Pasteur, Paris, France) as previously described^45^ and produced by two passages on Vero E6 cells in DMEM (Dulbecco’s Modified Eagles Medium) without FBS, supplemented with 1% P/S (penicillin at 10,000 U ml^−1^ and streptomycin at 10,000 μg ml^−1^) and 1 μg ml^−1^ TPCK-trypsin at 37 °C in a humidified CO_2_ incubator and titrated on Vero E6 cells. Whole genome sequencing was performed as described^45^ with no modifications observed compared with the initial specimen and sequences were deposited after assembly on the GISAID EpiCoV platform under accession number ID EPI_ISL_406596. Sequencing analysis revealed two clonal mutations, one in the *S* gene (22661G>T : V367F, non-synonymous) and one in the *ORF3a* gene (26144G>T : G251V, non-synonymous), which were already present in the challenge inoculum.

### Animals and study design

Seven week old female Balb/cJ mice (Jackson Laboratories Bar Harbor, ME) were anesthetized before being administered with 2.5 x 10^8^ PFU of human adenovirus type 5 encoding the human angiotensin converting enzyme-2 receptor (Ad5-hACE2) 5-days prior to challenge with SARS-CoV-2, as previously described^22,23^. Animals were transferred to the BSL-3 facility where two groups of n = 5 mice per group received 10 mg kg^−1^ of COVA1-18 intraperitoneally 24 h prior to, or post-infection with 10^4^ PFU SARS-CoV-2 in 50 μl PBS. A control group of n = 3 mice received 50 μl PBS. Mice were euthanized 3 d.p.i. and lungs harvested to quantify viral lung titers. Lungs were homogenized in PBS using a Beadblaster Microtube homogenizer (Benchmark Scientific). SARS-CoV-2 plaque assay was performed on 10-fold serial dilutions of lung homogenates prepared in 0.2% bovine serum albumin (BSA) in PBS that were plated onto a Vero E6 cells monolayer and incubated with shaking for 1 h. Inoculum was removed and plates were overlaid with Minimal Essential Media (MEM) containing 2% FBS/0.05% oxoid agar and incubated for 72 h at 37°C. Plates were fixed with 4% formaldehyde overnight, stained with a mAb cocktail composed of SARS-CoV-2 spike and SARS-CoV-2 nucleoprotein (Center for Therapeutic Antibody Discovery; NP1C7C7) followed by anti-Mouse IgG-HRP (Abcam ab6823) and developed using KPL TrueBlue peroxidase substrate (Seracare; 5510-0030).

Golden Syrian hamsters were randomly assigned to two groups of n = 5 and microchipped 24 h before SARS-CoV-2 challenge. On the day of challenge, hamsters were anesthetized with ketamine/xylazine and challenged by the intranasal route with 10^5^ PFU of SARS-CoV-2 diluted in sterile PBS in the total volume 100 μl. Body weight and body temperature were measured each day, starting at day 0. Twenty four hours post-challenge, hamsters were treated with 10 mg kg^−1^ of COVA1-18 diluted in 0.5 ml of sterile PBS via the intraperitoneal route. The control group of animals received an equal volume of sterile PBS via the intraperitoneal route. All animals were euthanized 72 h post-infection with an overdose of anesthetic (isoflurane or ketamine/xylazine) followed by bilateral thoracotomy, and terminal blood and lungs were collected at necropsy. Right lungs were frozen in 5 ml L-15 Leibowitz medium (Gibco) with 10% FBS. Tissue sections were homogenized in bead beater tubes, weighed, and supernatants were titrated per standard protocol. Briefly, of 10-fold dilutions of supernatants at 100 μl per well were placed atop of Vero-E6 monolayers in 96-well plates, the plates were incubated for 1 h, supernatants were replaced by methyl cellulose overlay, incubated for 3 days at 5% CO_2_ and 37 °C. The plates were fixed with formalin, removed from BSL-4 according the approved protocol, and plaques counted to determine the viral titers.

Ten female cynomolgus macaques were randomly assigned between the control and treated groups to evaluate the efficacy of COVA1-18 prophylaxis. The treated group (n = 5) received one bolus dose of COVA-18 human IgG1 monoclonal antibody (10 mg kg^−1^) by the intravenous route in the saphenous vein one day prior to challenge, while control animals (n = 5) received no treatment. All animals were then exposed to a total dose of 10^6^ PFU of SARS-CoV-2 (BetaCoV/France/IDF/0372/2020; passaged twice in VeroE6 cells) *via* the combination of intranasal and intratracheal routes (day 0), using atropine (0.04 mg kg^−1^) for pre-medication and ketamine (5 mg kg^−1^) with medetomidine (0.05 mg kg^−1^) for anesthesia. Animals were observed daily and clinical exams were performed at baseline, daily for one week, and then twice weekly, on anaesthetized animals using ketamine (5 mg kg^−1^) and metedomidine (0.05 mg kg^−1^). Body weight and rectal temperature were recorded and blood, as well as nasopharyngeal, tracheal and rectal swabs, were collected. Broncho-alveolar lavages (BAL) were performed using 50 ml sterile saline on 3 d.p.i. Chest CT was performed at 3 d.p.i. in anesthetized animals using tiletamine (4 mg kg^−1^) and zolazepam (4 mg kg^−1^). Blood cell counts, haemoglobin and haematocrit were determined from EDTA blood using a DHX800 analyzer (Beckman Coulter).

One male and one female cynomolgus macaques received the treatment as described above for the pharmacokinetic and pharmacodynamics (PK/PD) study. Blood was sampled before and 2, 4, 6 and 24 h post-treatment. Saliva, nasopharyngeal and tracheal fluids were sampled before and 24 h post-treatment. Twenty-four hours post-treatment, animals were euthanized and their lungs, heart, kidney, liver, spleen, trachea and brain were sampled, rinsed with PBS and around 100 mg of tissue was homogenized in 500 μl of PBS with a Precellys and stored at −80°C.

### Virus quantification in NHP samples

Upper respiratory (nasopharyngeal and tracheal) and rectal specimens were collected with swabs (Viral Transport Medium, CDC, DSR-052-01). Tracheal swabs were performed by insertion of the swab above the tip of the epiglottis into the upper trachea at approximately 1.5 cm of the epiglottis. All specimens were stored between 2°C and 8°C until analysis by RT-qPCR with a plasmid standard concentration range containing an RdRp gene fragment including the RdRp-IP4 RT-PCR target sequence. SARS-CoV-2 E gene subgenomic mRNA (sgRNA) levels were assessed by RT-qPCR using primers and probes previously described^46,47^: leader-specific primer sgLeadSARSCoV2-F CGATCTCTTGTAGATCTGTTCTC, E-Sarbeco-R primer ATATTGCAGCAGTACGCACACA and E-Sarbeco probe HEX-ACACTAGCCATCCTTACTGCGCTTCG-BHQ1. The protocol describing the procedure for the detection of SARS-CoV-2 is available on the WHO website (https://www.who.int/docs/default-source/coronaviruse/real-time-rt-pcr-assays-for-the-detection-of-sars-cov-2-institut-pasteur-paris.pdf?sfvrsn=3662fcb6_2).

### Chest CT and image analysis

Lung images were acquired using a computed tomography (CT) system (Vereos-Ingenuity, Philips) as previously described^25,26^. Lesions were defined as ground glass opacity, crazy-paving pattern, consolidation or pleural thickening as previously described^32,48^. Lesions and scoring were assessed in each lung lobe blindly and independently by two persons and the final results were established by consensus. Overall CT scores include the lesion type (scored from 0 to 3) and lesion volume (scored from 0 to 4) summed for each lobe as previously described^25,26^.

### Viral sequencing

10 RNA samples from nasopharyngeal swabs at day 3 post-exposure were selected for sequencing along with the inoculum. cDNA and multiplex PCR reactions were prepared following the ARTIC SARS-CoV-2 sequencing protocol v2^49^. V3 primer scheme (https://github.com/artic-network/primer-schemes/tree/master/nCoV-2019/V3) was used to perform the multiplex PCR for SARS-CoV-2. All samples were run for 35 cycles in the two multiplex PCRs. Pooled and cleaned PCR reactions were quantified using QubitTM fluorometer (Invitrogen). The Ligation Sequencing kit (SQK-LSK109; Oxford Nanopore Technologies) was used to prepare the library following the manufacturer’s protocol ("PCR tiling of COVID-19 virus", release F; Oxford Nanopore Technologies). Twenty-four samples were multiplexed using Native Barcoding Expansion 1-12 and Native Barcoding Expansion 13-24 kits (EXP-NBD104 and EXP-NBD114; Oxford Nanopore Technologies). Two libraries of 24 samples were prepared independently and quantified by QubitTM fluorometer (Invitrogen). After the quality control, two R9.4 flowcells (FLO-MIN106; Oxford Nanopore Technologies) were primed as described in the manufacturer’s protocol and loaded with 45 and 32 ng of library. Sequencing was performed on a GridION (Oxford Nanopore Technologies) for 72h with high-accuracy Guppy basecalling (v3.2.10). After sequencing, demultiplexing was performed using Guppy v4.0.14 with the option --require_barcodes_both_ends to ensure high quality demultiplexing. Reads were then filtered by Nanoplot v1.28.1 based on length and quality to select high quality reads. Then, reads were aligned on the SARS-CoV-2 reference genome NC_045512.2 using minimap2 v2.17. Primary alignments were filtered based on reads length alignment and reads identity. Reads were basecalled and demultiplexed with Guppy 4.0.14. The potential clonal and subclonal variants were detected with a custom pipeline based on ARTIC network workflow. Longshot v0.4.1 was used for variant detection. The potential subclonal variants were manually curated by comparing the generated VCF files and visual inspection of the alignments in IGV browser.

### Statistical analysis

Statistical analysis of Syrian hamsters and hACE2 mice lung viral titers as well as for NHP gRNA and sgRNA were carried out using Mann-Whitney unpaired t-test in GraphPad Prism software (v8.3.0).

## Extended Data

**Extended Data Table 1.** BLI and neutralization potency of IgG vs Fab in HEK293T hACE2 cells. AMC and Duke neutralization assays use lentiviral pseudotyped particles and HEK293T hACE2 cells. Nexelis neutralization assay uses VSVΔG pseudotyped particles and Vero E6 cells. BLI, biolayer interferometry; RBD, receptor binding domain.

		IC_50_					BLI					
		AMC (n ≥ 4)		Duke (n = 1)	Duke D614G (n = 1)	Nexelis (n = 1)	RBD loaded (n = 3)			Soluble S loaded (n = 3)		
		ng ml^−1^	pM	ng ml^−1^			K_D_ (nM)	Ka (M^−1^s^−1^)	Kd (s^−1^)	K_D_ (nM)	Ka (M^−1^s^−1^)	Kd (s^−1^)
**1-18**	**IgG**	0.8	5.6	9.0	7.0	9.0	7.0	1.7E+05	1.3E-03	5.0	3.7E+05	1.9E-03
	**Fab**	199.0	3968.0	N/A	N/A	N/A	84.1	5.0E+04	4.1E-03	N/A	N/A	N/A

**Extended Data Table 2.** Parameter estimates of the viral dynamic model. RSE: relative standard error

Parameters	Unit	Description	Fixed effect (RSE%)	Sd of random effect (RSE%)
β_N_	ml per copies per day	Virion infectivity	2.14×10^−4^	0.378
β_T_	ml per copies per day		1.68×10^−3^	
p_N_	copies d^−1^	Viral production	3.32×10^4^	0.552 (99.3)
p_T_	copies d^−1^		1.12×10^4^	
*f*		Scaling factor for the subgenomic RNA	6.98 (66.7)	1.53 (36)
EC_50N_	μg ml^−1^	Concentration required to block infectivity by 50%	2.2	0.366 (133)
EC_50T_	μg ml^−1^		0.053	
EC_90N_	μg ml^−1^	Concentration required to block infectivity by 90%	19.8	
EC_90T_	μg ml^−1^		0.48	
*δ*	d^−1^	Baseline clearance rate of productively infected cells	1.88	0.172
*ka*	d^−1^	Absorption rate	4.45	
*k*	d^−1^	Elimination rate	0.0549 (13.3)	0.225 (44.5)
*V*	ml kg^−1^	Volume of distribution	88.7 (6.56)	
*D*	mg kg^−1^	Administered dose of COVA1-18	10	

**Extended Data Table 3.** Mean individual efficacy of the COVA1-18 for each individual in both compartments (calculated over the first 10 days of administration).

		MF6	MF7	MF8	MF9	MF10
Mean efficacy (%)	Nasopharynx	97.34	96.67	97.37	97.50	97.16
	Trachea	99.93	99.91	99.93	99.94	99.93

## Supplementary Material

Supplement

## References

[1] Salama C. Tocilizumab in Patients Hospitalized with Covid-19 Pneumonia. N. Engl. J. Med. (2020) doi:10.1056/nejmoa2030340 33657287

[2] Horby P. Effect of Dexamethasone in Hospitalized Patients with COVID-19: Preliminary Report. N. Engl. J. Med. (2020) doi:10.1101/2020.06.22.20137273 PMC7383595 32678530

[3] Update to living WHO guideline on drugs for covid-19. BMJ (2020) doi:10.1136/bmj.m4475 33214213

[4] Weinreich D. M. REGN-COV2, a Neutralizing Antibody Cocktail, in Outpatients with Covid-19. N. Engl. J. Med. 384, (2021). 10.1056/NEJMoa2035002 PMC7781102 33332778

[5] Chen P. SARS-CoV-2 Neutralizing Antibody LY-CoV555 in Outpatients with Covid-19. N. Engl. J. Med. 384, (2021). 10.1056/NEJMoa2029849 PMC7646625 33113295

[6] A Neutralizing Monoclonal Antibody for Hospitalized Patients with Covid-19. N. Engl. J. Med. NEJMoa2033130 (2020) doi:10.1056/NEJMoa2033130 PMC7781100 33356051

[7] Brouwer P. J. M. Potent neutralizing antibodies from COVID-19 patients define multiple targets of vulnerability. Science 369, (2020). 10.1126/science.abc5902 PMC7299281 32540902

[8] Rees-Spear C. The impact of Spike mutations on SARS-CoV-2 neutralization. Preprint at 10.1101/2021.01.15.426849 PMC7936541 33713594

[9] Shen X. SARS-CoV-2 variant B.1.1.7 is susceptible to neutralizing antibodies elicited by ancestral Spike vaccines. Preprint at 10.1101/2021.01.27.428516 PMC7934674 33705729

[10] COVID-19 Dashboard by the Center for Systems Science and Engineering (CSSE) at Johns Hopkins University (JHU). https://coronavirus.jhu.edu/map.html (2021). 10.1016/S1473-3099(22)00434-0 PMC9432867 36057267

[11] Zost S. J. Rapid isolation and profiling of a diverse panel of human monoclonal antibodies targeting the SARS-CoV-2 spike protein. Nat. Med. 26, (2020). 10.1038/s41591-020-0998-x PMC8194108 32651581

[12] Cao Y. Potent Neutralizing Antibodies against SARS-CoV-2 Identified by High-Throughput Single-Cell Sequencing of Convalescent Patients’ B Cells. Cell 182, (2020). 10.1016/j.cell.2020.05.025 PMC7231725 32425270

[13] Hansen J. Studies in humanized mice and convalescent humans yield a SARS-CoV-2 antibody cocktail. Science 369, (2020). 10.1126/science.abd0827 PMC7299284 32540901

[14] Wu Y. A noncompeting pair of human neutralizing antibodies block COVID-19 virus binding to its receptor ACE2. Science 368, (2020). 10.1126/science.abc2241 PMC7223722 32404477

[15] Schmidt F. Measuring SARS-CoV-2 neutralizing antibody activity using pseudotyped and chimeric viruses. J. Exp. Med. 217, (2020). 10.1084/jem.20201181 PMC7372514 32692348

[16] Almahboub S. A., Algaissi A., Alfaleh M. A., ElAssouli M.-Z. & Hashem A. M. Evaluation of Neutralizing Antibodies Against Highly Pathogenic Coronaviruses: A Detailed Protocol for a Rapid Evaluation of Neutralizing Antibodies Using Vesicular Stomatitis Virus Pseudovirus-Based Assay. Front. Microbiol. 11, (2020). 10.3389/fmicb.2020.02020 PMC7498578 33013745

[17] Korber B. Tracking Changes in SARS-CoV-2 Spike: Evidence that D614G Increases Infectivity of the COVID-19 Virus. Cell 182, (2020). 10.1016/j.cell.2020.06.043 PMC7332439 32697968

[18] Yurkovetskiy L. Structural and Functional Analysis of the D614G SARS-CoV-2 Spike Protein Variant. Cell 183, (2020). 10.1016/j.cell.2020.09.032 PMC7492024 32991842

[19] Plante J. A. Spike mutation D614G alters SARS-CoV-2 fitness. Nature (2020) doi:10.1038/s41586-020-2895-3 PMC8158177 33106671

[20] Mansbach R., Chakraborty S., Nguyen K., Montefiori D. & Korber B. The SARS-CoV-2 Spike Variant D614G Favors an Open Conformational State. Preprint at 10.1101/2020.07.26.219741 PMC8051874 33863729

[21] Volz E. Evaluating the Effects of SARS-CoV-2 Spike Mutation D614G on Transmissibility and Pathogenicity. Cell (2020) doi:10.1016/j.cell.2020.11.020 PMC7674007 33275900

[22] Hassan A. O. A SARS-CoV-2 Infection Model in Mice Demonstrates Protection by Neutralizing Antibodies. Cell 182, (2020). 10.1016/j.cell.2020.06.011 PMC7284254 32553273

[23] Rathnasinghe R. Comparison of transgenic and adenovirus hACE2 mouse models for SARS-CoV-2 infection. Emerg. Microbes Infect. 9, 2433–2445 (2020). 33073694 10.1080/22221751.2020.1838955 PMC7655046

[24] Muñoz-Fontela C. Animal models for COVID-19. Nature vol. 586 509–515 (2020). 32967005 10.1038/s41586-020-2787-6 PMC8136862

[25] Maisonnasse P. Hydroxychloroquine use against SARS-CoV-2 infection in non-human primates. Nature 585, 584–587 (2020). 32698191 10.1038/s41586-020-2558-4

[26] Brouwer P. J. M. Two-component spike nanoparticle vaccine protects macaques from SARS-CoV-2 infection. Cell 2020.11.07.365726 (2021) doi:10.1016/j.cell.2021.01.035 PMC7834972 33577765

[27] Andreano E. SARS-CoV-2 escape &lt;em&gt;in vitro&lt;/em&gt; from a highly neutralizing COVID-19 convalescent plasma. Preprint at doi:10.1101/2020.12.28.424451 PMC8433494 34417349

[28] Gonçalves A. Viral dynamic modeling of SARS-CoV-2 in non-human primates. Res. Sq. (2021) doi:10.21203/rs.3.rs-50301/v1

[29] Baum A. REGN-COV2 antibodies prevent and treat SARS-CoV-2 infection in rhesus macaques and hamsters. Science (2020) doi:10.1126/science.abe2402 PMC7857396 33037066

[30] Zost S. J. Potently neutralizing and protective human antibodies against SARS-CoV-2. Nature 584, (2020). 10.1038/s41586-020-2548-6 PMC7584396 32668443

[31] Jones B. E. Title: LY-CoV555, a rapidly isolated potent neutralizing antibody, provides protection in a non-human primate model of SARS-CoV-2 infection. doi:10.1101/2020.09.30.318972

[32] Shi R. A human neutralizing antibody targets the receptor-binding site of SARS-CoV-2. Nature 584, (2020). 10.1038/s41586-020-2381-y 32454512

[33] Wang S. Characterization of neutralizing antibody with prophylactic and therapeutic efficacy against SARS-CoV-2 in rhesus monkeys. Nat. Commun. 11, (2020). 10.1038/s41467-020-19568-1 PMC7666115 33188207

[34] Li D. The functions of SARS-CoV-2 neutralizing and infection-enhancing antibodies in vitro and in mice and nonhuman primates. Preprint at 10.1101/2020.12.31.424729

[35] Fagre A. C. A Potent SARS-CoV-2 Neutralizing Human Monoclonal Antibody That Reduces Viral Burden and Disease Severity in Syrian Hamsters. Front. Immunol. 11, (2020). 10.3389/fimmu.2020.614256 PMC7775388 33391285

[36] Wu K. mRNA-1273 vaccine induces neutralizing antibodies against spike mutants from global SARS-CoV-2 variants. Preprint at 10.1101/2021.01.25.427948

[37] Wang P. Increased Resistance of SARS-CoV-2 Variants B.1.351 and B.1.1.7 to Antibody Neutralization. Preprint at 10.1101/2021.01.25.428137

[38] Starr T. N. Prospective mapping of viral mutations that escape antibodies used to treat COVID-19. Science (2021) doi:10.1126/science.abf9302 PMC7963219 33495308

[39] Wibmer C. K. SARS-CoV-2 501Y.V2 escapes neutralization by South African COVID-19 donor plasma. Preprint at 10.1101/2021.01.18.427166 33654292

[40] Czuppon P. Success of prophylactic antiviral therapy for SARS-CoV-2: predicted critical efficacies and impact of different drug-specific mechanisms of action. Preprint at 10.1101/2020.05.07.20092965 PMC7951973 33647008

[41] Néant N. Modeling SARS-CoV-2 viral kinetics and association with mortality in hospitalized patients: results from the French Covid-19 cohort. Proc Natl Acad Sci U S A (2021). 10.1073/pnas.2017962118 PMC7929555 33536313

[42] Baum A. Antibody cocktail to SARS-CoV-2 spike protein prevents rapid mutational escape seen with individual antibodies. Science 369, (2020). 10.1126/science.abd0831 PMC7299283 32540904

[43] Saunders K. O. Conceptual Approaches to Modulating Antibody Effector Functions and Circulation Half-Life. Front. Immunol. 10, (2019). 10.3389/fimmu.2019.01296 PMC6568213 31231397

