# Supplementary material for: COVA1-18 neutralizing antibody protects against SARS-CoV-2 in three preclinical models
Source: Res Sq. 2021 Feb 15:rs.3.rs-235272. Preprint. [Version 1] doi: 10.21203/rs.3.rs-235272/v1 (PMC7899470; doi:10.21203/rs.3.rs-235272/v1)
Supplement: Supplement [file 58ca15a9f9c6ae330311234f.docx]

**Supplementary information:**

**Methods**

**Mouse experiment viruses**

*Ad5-hACE2 production.* A seed stock for an E1/E3 deleted, non-replicating adenoviral vector based on human adenovirus type 5 (Ad5), encoding the human angiotensin converting enzyme-2 receptor (hACE2) under the control of a CMV promoter, was obtained from Iowa Viral Vector Core Facility. High titer stocks of Ad5-hACE2 were generated by amplification in T-Rex™-293 cells (Life Technologies, Carlsbad, CA), and purification with two rounds of cesium chloride (CsCl) ultracentrifugation. Virus was titered on T-Rex™-293, using the tissue culture infectious dose-50 (TCID50) endpoint dilution method with titers adjusted to PFU ml^-1^ as described previously^50,51^.

*SARS-CoV-2:* Human isolate USA-WA1/2020 (BEI resources; NR-52281) was propagated in Vero E6 cells under BSL-3 containment in accordance with the biosafety protocols developed by the Icahn School of Medicine at Mount Sinai. Viral stocks were grown in Dulbecco Modified Eagle’s Medium containing 2% fetal bovine serum, 2-[4-(2-hydroxyethyl)piperazin-1-yl]ethanesulfonic acid (HEPES), and penicillin–streptomycin for 72 h and were validated by genome sequencing. Cells were infected at a multiplicity of infection (MOI) of 0.1; mice were infected with 1×10^4^ PFU.

**Viral kinetic model (VK)**

We used a previously published model characterizing nasopharyngeal and tracheal SARS-CoV-2 infection kinetics^28^. In this model, nasopharynx and trachea are modeled as two separated compartments described by a target cell limited model^52–54^ as follows:

$$\frac{dT^{x}}{dt}=-\beta^{x}T^{x}V_{I}^{x} (1)$$

$$\frac{dI_{1}^{x}}{dt}=\beta^{x}T^{x}V_{I}^{x}-kI_{1}^{x} (2)$$

$$\frac{dI_{2}^{x}}{dt}=kI_{1}^{x} -\delta I_{2}^{x} (3)$$

$$\frac{dV_{I}^{x}}{dt}=p^{x}\mu I_{2}^{x}-cV_{I}^{x} (4)$$

$$\frac{dV_{NI}^{x}}{dt}=p^{x}\left( 1-\mu\right)I_{2}^{x}-cV_{NI}^{x} (5)$$

Where $T, I_{1}, I_{2}, V_{I} and V_{NI}$ are the densities of target cells, infected non-productive cell, infected productive cell, infectious virus and non-infectious virus respectively and the subscript $x$ denotes the compartment of interest, either nasopharynx or trachea. Target cells ($T$) are infected at a rate $\beta$ by infectious virus ($V_{I}$). Infected cells enter an eclipse phase ($I_{1}$) where they do not produce virions before becoming productively infected cells ($I_{2}$) at rate $k$. $I_{2}$ cells then produce virions at a rate $p$ per days and are lost at a rate $\delta$. A proportion $\mu$ of produced virions are infectious ($V_{I}$) and the remaining $(1-\mu)$ are non-infectious viruses ($V_{NI}$), both cleared at a rate $c$.

We assumed that subgenomic RNA,$V_{sg}^{x}$ , is a proxy for the number of infected cells, and we wrote $V_{sg}^{x}=f\left( I_{1}^{x}+I_{2}^{x} \right) (6)$

Where $f$ is a scaling factor between sgRNA and the number of infected cells.

**COVA1-18 pharmacokinetics model (PK)**

We made use of a pharmacokinetic model with one compartment and a linear elimination to describe the dynamics of the COVA1-18 neutralizing antibody. The plasma concentration is given by the following analytical solution^55^:

$$C\left( t \right)= \frac{D}{V}\frac{ka}{ka-k}\left( e^{-k\left( t-t_{d} \right)}-e^{-ka\left( t-t_{d} \right)} \right) (7)$$

Where $D, V, k, ka and t_{d}$ are the dose administered, the volume of distribution, the elimination rate, the absorption rate and the initiation of treatment, respectively. As the absorption phase could not be estimated, we fixed k_a_ $=4.45 d^{-1}$, which corresponds to a $T_{max}$ of 1 $d$, as observed in the data.

**Concentration-Effect relationship (PK/PD)**

To describe the interaction between the neutralizing antibody concentration and its efficacy, we used an $E_{max}$ model, where the efficacy is linked to the antibody plasma concentration as:

${\eta(t)}^{x}=\frac{C(t)}{{EC}_{50}^{x}+C(t)} (8)$

Where ${EC}_{50}$ is the plasma concentration of COVA1-18 required to reduce infectivity by 50%. Because the drug concentration, and hence the drug efficacy, is time-dependent, we calculated the mean individual efficacy over the first 10 days of infection as given by $\eta_{mean}^{x}=\int_{t=0}^{10} {\eta(u)}^{x}du$

**Fixed parameters**

We used the same assumptions as in ^28^.We fixed the $T_{N}\left( t=0 \right)=1.25\times{10}^{5}$ and $T_{T}\left( t=0 \right)=2.25\times{10}^{4}$ cells in nasopharynx and trachea respectively. Second, we assumed the proportion of infectious viruses $\mu$ remained constant over time and equal to ${10}^{-4}$.Third, we fixed the eclipse phase duration to $k=3 {day}^{-1}$ and the viral clearance $c=10 d^{-1}$.

**Parameter estimation and statistical methods**

Parameter estimation was performed using maximum-likelihood methods using the stochastic approximation expectation-maximisation (SAEM) algorithm implemented in MONOLIX^56,57^.

The PK/PD model was build using a two-stage approach. We fitted the PK data and injected the individual estimated parameters in the VK model. The estimated parameters for the PK models were $V$ and $k$, and the estimated parameters for the VK model were $f,{EC}_{50}^{T}, {EC}_{50}^{N}, p^{T}and p^{N}$.

**Simulation settings**

The model was used to evaluate the effects of different experimental setting, such as the virus infectious dose, the timing of treatment initiation, or the dose of COVA1-18. Using the parameters given in Extended Data Table 3, we simulated different experimental designs and observed the predicted viral load (Genomic and Subgenomic) for both nasopharynx and trachea compartments. Several scenarios were assessed, according to inoculum size (${10}^{4}, {10}^{5} and {10}^{6} PFU),$COVA1-18 dose ($0.1, 1, 5, and 10 mg per kg)$, and timing of treatment initiation ($-1 d.p.i.+1 d.p.i.)$. The simulations were performed using the Simulx package on R.3.6.0.
